# Editorial: Mentalization and clinical psychopathology, volume II

**DOI:** 10.3389/fpsyg.2024.1424088

**Published:** 2024-06-04

**Authors:** Vanya L. Matanova, Drozdstoy S. Stoyanov, Olga Strizhitskaya

**Affiliations:** ^1^Institute of Mental Health and Development, Sofia, Bulgaria; ^2^Department of Psychiatry and Medical Psychology, Medical University, Plovdiv, Bulgaria; ^3^Research Institute, Medical University, Plovdiv, Bulgaria; ^4^Department of Developmental and Differential Psychology, Saint-Petersburg State University, Saint Petersburg, Russia

**Keywords:** mentalization, psychopathology, psychotherapy, wellbeing, trauma

Mentalization originates from psychodynamic thinking, natural pedagogy, systematic approach, and cognitive and behavioral therapies. Mentalization-based therapy links many clinical symptoms to the manifesting characteristics of attachment systems. Mentalization is an individual's capacity to imagine and understand others and ourselves (Fonagy and Allison, 2013). It has cognitive and affective components (Fonagy and Target, 2006), and we can suggest that it is somehow associated with reflective functioning (Martin-Gagnon et al.). Mentalization is closely related to developmental processes and can be considered a developmental achievement (Ensink and Mayes, 2010); in other words, the process of development itself determines the quality of mentalization. It is safe to assume that better developmental conditions would predict better mentalization and that trauma would negatively affect this process (Ensink et al., 2015). Some studies argue that mentalization is a resilience factor (Borelli et al., 2021). Thus the nature, mechanisms, and effects of mentalization remain unclear and need further investigation.

This Research Topic uncovers a variety of approaches and applications to mentalization. We highlight the richness of the topic and its importance for the future of psychotherapy and research. We have tried to reflect on mentalization from structural, methodological, and applied perspectives and to show how it can be incorporated into the treatment of different psychopathologies.

In this Research Topic, we approached mentalization from theoretical, empirical, and applied perspectives. Five various articles were included, and all of them addressed the analysis, research of mentalization, its effects, potential, and place in the system of psychotherapy approaches. The theoretical perspective is described in this Research Topic by Arabadzhiev and Paunova. It showed mentalization as a complex phenomenon and described its components, levels, and effects. The authors argued that mentalization is critical for a variety of relationships that an individual is involved in. Therefore, it is essential to understand the phenomenon at biological, psychological, and social levels, as this is an essential part of supporting people who have problems with mentalization in psychotherapy.

The articles by Martin-Gagnon et al. investigated the associations between mentalization, childhood abuse, anxiety, depression, and borderline disorders. In their Research Topic, they confirmed the mentalization process of uncertainty and confusion, playing an important role in well-established associations between childhood abuse and depression (Humphreys et al., 2020), anxiety (Guo et al., 2021), and borderline disorders (Porter et al., 2020).

Two articles broaden the understanding of mentalization within the psychotherapy process. One of the articles describes the complexity of the effects of trauma, particularly its long-term effects and translational patterns of transgenerational trauma. It highlights the variety of outcomes that trauma can have for future generations and approaches to dealing with it (Kostova and Matanova). Another article describes the role of play in adolescents' lives and discusses it as a potential instrument for therapy (Kolev). The author highlights that while play can be viewed as a basic mechanism and even an intrinsic need for teenagers and adolescents, we should be very sensitive and accurate in applying play to a therapeutical process.

Finally, one article suggests a novel, and, to some extent, a revolutionary approach to understanding cognitive biases in autism and schizophrenia (Rządeczka et al.). The authors argue that some types of autism and schizophrenia that are traditionally viewed as pathologies could be specific mental forms of adaptation for our ancestors. From this evolutionary perspective, specific cognitive strategies that have a poor fit for contemporary society could have been useful or even advantageous at earlier evolutionary stages.

The scope of the present topic uncovers a wide range of applications of mentalization. The idea of mentalization originates from therapy and has shown its benefits and potential over the past couple of decades. In this Research Topic, we showed that mentalization attracts more interest than just a therapy method. It promoted theoretical and empirical research for a better understanding of it.

In conclusion, although our topic focuses on mentalization and psychopathology, it also opens a perspective to investigate and apply it in a broader context. While in psychopathology, mentalization can be an effective instrument for dealing with a variety of issues and disorders, we can also assume that its mechanisms could assist a better understanding of normal mental functioning. We could expect that further investigation of mentalization could help in understanding intergenerational relationships and resolving the developmental task of dealing with self-determination and other processes that are crucial for one's mental wellbeing.

## Author contributions

VM: Writing – review & editing, Methodology, Conceptualization. DS: Writing – review & editing, Project administration, Methodology, Conceptualization. OS: Writing – review & editing, Writing – original draft, Conceptualization.
